# Handedness and Relative Age in International Elite Interactive Individual Sports Revisited

**DOI:** 10.3389/fspor.2021.662203

**Published:** 2021-03-31

**Authors:** Florian Loffing, Jörg Schorer

**Affiliations:** Research Group “Sport and Movement Science”, Institute of Sport Science, Carl von Ossietzky University of Oldenburg, Oldenburg, Germany

**Keywords:** racket sports, birthdate, laterality, season of birth, birthday, talent development, youth

## Abstract

Relative age effects (RAE) describe the unintended side effect of annual age grouping such that athletes born close to a specific cutoff date are more likely to be associated with attaining higher performance status than athletes born later. One factor suggested to override the RAE is handedness. Given the left-handers' rarity and their proposed performance advantage in interactive sports, left-handedness may be associated with a lower likelihood of suffering from selection inequalities like RAE in those sports compared with right-handedness. Here, in a two-study approach, we tested that hypothesis by examining male and female athletes from various interactive individual sports sampled over a 10-year period from 2007 to 2016. Study 1 investigated distributions of birth and handedness of senior athletes listed in the top 200 of year-end world rankings in table tennis, tennis, squash, and fencing (épée, foil, and saber). Study 2 followed a similar design but focused on junior athletes in the fencing disciplines and tennis. Unlike the above prediction, in both studies, birth distribution was not found to be reliably associated with handedness in any of the sports or disciplines considered. Left-handers were consistently overrepresented in épée, foil, and table tennis, occasionally in saber and tennis, and not at all in squash. Birth frequencies decreased from quartile Q1 (January to March) to Q4 in almost any sporting domain at the junior level, whereas such trend was rarely found at the senior level. In conclusion, while providing novel insight on the role handedness may play at the junior level, our findings do not support the hypothesis that left-handedness helps override birth-related inequalities in high sporting achievement in elite interactive individual sports.

## Introduction

Once you are born at the right time, you have it in your own hands to reach elite levels in interactive individual sports, or so it seems (Deaner et al., 2013). However, one's own birthdate and laterality independently seem to be associated with different probabilities to become an elite athlete (Loffing et al., 2010; Barrenetxea-Garcia et al., 2019). Much less is known about the interaction of these two moderators (Wattie et al., 2015). Here, we revisit the association of relative age and handedness with achieving world-class performances in interactive sports in adult and junior athletes.

In many interactive (as well as others) sports, sport policy makers decided to use a cutoff date like the first of January to reduce injustices in competitions (Dixon et al., 2020). While this was well intended, it still resulted in relative age effects (RAE), in which athletes born closer to the cutoff date had a higher probability to make it than others that were born later in the year. The effect was reported in a wide range of interactive individual sports like tennis (Edgar and O'Donoghue, 2005; O'Donoghue, 2009), table tennis (Faber et al., 2020a), and fencing (Romann and Fuchslocher, 2014; Romann et al., 2018; Switzerland), but not in English squash (Kelly et al., 2020a), and was generally stronger in male than in female athletes (Smith et al., 2018). In sports, the RAE phenomenon increases with age, and it reaches its peak during adolescence (15–18 years) and reduces at the senior level (Cobley et al., 2009). Moreover, according to the competition hypothesis, the likelihood for and the size of RAE increase with increasing competition for the limited resources (e.g., squad membership) in a sport (Sherar et al., 2007). Musch and Grondin (2001) considered competition a necessary condition for RAE to occur at all.

After 30 years of research on the topic, finally, a first theoretical model was introduced by Wattie et al. (2015). On the basis of the work by Bronfenbrenner and colleagues (Bronfenbrenner and Ceci, 1994; Bronfenbrenner, 2000), Wattie and colleagues presented a developmental model of RAE. The idea of Newell's (1986) constraint-based developmental model results in a framework that differentiates influential variables in three different constraints: environmental, task, and individual constraints. As environmental constraints, the authors summarize variables like policies, social norms, quality of sport development programs, and family influences (Wattie et al., 2007; Cobley et al., 2008; Addona and Yates, 2010; Hancock et al., 2013; Schorer et al., 2013). As task constraints, they introduce the type of sport and level of competition (Schorer et al., 2009; Smith et al., 2018; Faber et al., 2020a). As individual constraints, variables like sex (Smith et al., 2018) or laterality (Loffing et al., 2010; Barrenetxea-Garcia et al., 2019) are considered. While some constraints have been given a considerable amount of attention, little is known about the interaction of laterality and RAE (Wattie et al., 2015).

The rarity of left- compared with right-handers is suggested to provide the former with a performance advantage in a variety of interactive sports (for reviews, e.g., see Grouios, 2004; Loffing and Hagemann, 2012, 2016). Athletes performing a sport-specific task with the left hand (e.g., holding a racket or weapon) have consistently been found disproportionally more often compared with normal population estimates of left-handedness at the top level of interactive individual sports like table tennis (Malagoli Lanzoni et al., 2013; Loffing, 2017) and the fencing disciplines of foil and épée, but not saber (Raymond et al., 1996; Harris, 2016). These findings suggest that handedness is not a neutral trait with regard to performance in these sports, especially not in male competition where a left-hander overrepresentation was found more pronounced compared with female competition (Raymond et al., 1996; Breznik, 2013; Loffing, 2017). As a caveat to this suggestion, however, left-handers were only occasionally found overrepresented in professional tennis (Holtzen, 2000; Loffing et al., 2012) and not at all in squash (Loffing, 2017), which indicates that a left-hander advantage may be particularly evident in interactive sports that are characterized by high spatiotemporal pressure (e.g., table tennis, fencing) as opposed to relatively slower interactive sports (e.g., tennis, squash) (Loffing, 2017).

In view of the performance benefits ascribed to left-handedness in interactive sports, it has been suggested that left-handedness may be associated with reduced likelihood of suffering from selection inequalities like RAE (Wattie et al., 2015). Evidence in at least partial support of this notion can primarily be inferred from data on interactive team sports like handball (Schorer et al., 2009), cricket (Connor et al., 2019), baseball (Zhang et al., 2018), and water polo (Barrenetxea-Garcia et al., 2019). Birth distribution was found to be heavily skewed toward quartile 1 (January–March) in right- but not left-handed backcourt players from the German first handball league for the seasons 2004/2005–2007/2008 (Schorer et al., 2009). Published research examining the possible link between RAE and handedness in individual interactive sports, however, is surprisingly scarce. The only study available to date investigated RAE and handedness in a sample of 1,027 male professional senior tennis players (13.44% left-handed) listed in the top 500 of year-end world rankings in 2000–2006 (Loffing et al., 2010). Birth distribution was skewed toward quartiles 1 (January–March) and 2 (April–June) in right- but not in left-handed players. The effect of left-handed players being born more often in the second (52.9%) than in the first half of the year and a reversed pattern of birth frequencies in right-handed players (first half: 57.59%), however, was very small [*w* = 0.07, 90% CI (0.02, 0.12)]. Overall, given the paucity of substantial evidence in favor of a reliable association between handedness and RAE, it remains open whether laterality, and handedness in particular, is not only a statistical but also a practically meaningful factor for explaining the RAE phenomenon in sports (Wattie et al., 2015).

The overarching aim of this work was to close this research gap. In a two-study approach, we first investigated the association of RAE and handedness in varying interactive sports at the senior level. In a second study, the association was tested further in junior athletes of interactive sports. Both age groups were included given the clear indication that the extent of RAE varies with age (Cobley et al., 2009). Overall, the two studies were intended to provide us with a clearer picture of the potential association between relative age and handedness.

## Study 1: Relative Age Effects and Handedness in World-Class Senior Athletes

Here, we sought to test the hypothesis that top-ranked athletes' left-handedness is associated with lower likelihood of suffering the commonly observed RAE phenomenon as compared with right-handedness in individual interactive sports at the senior level (cf. Schorer et al., 2009; Loffing et al., 2010; Barrenetxea-Garcia et al., 2019). If so, birth distribution should be skewed more heavily toward the “classical” RAE pattern in right-handed as opposed to left-handed athletes. To enhance the generalizability of the findings across different individual interactive sports where left-handers are suggested to be advantaged (e.g., table tennis, fencing) or rather not (e.g., squash, tennis) (Raymond et al., 1996; Loffing, 2017) as well as to account for potential temporal variability in the RAE phenomenon (O'Donoghue, 2009; Schorer et al., 2020), we considered data on 10 year-end world rankings from 2007 to 2016 in six different interactive sports or sporting disciplines [fencing (épée, foil, saber), table tennis, tennis, squash].

## Materials and Methods

### Data Retrieval

The lists of senior female and male top 200 athletes in various sports' year-end world rankings from 2007 to 2016 were manually retrieved from the sports' official websites (Table 1). Athletes' task-specific handedness (i.e., hand used for holding a fencing weapon or a racket) was assessed from the same websites or determined based on additional searches on the web such as pictures or videos showing an athlete in action. The full raw dataset underlying this study is made available in the accompanying Supplementary Material.

**Table 1:** Web sources used for the retrieval of year-end world rankings in 2007 to 2016 and total number of unique female and male senior (study 1) and junior (study 2) athletes.

**Age group**	**Sport (discipline)**	***N*_**female**_**	***N*_**male**_**	**Source**
Senior (study 1)	Fencing (épée)	629	652	www.fie.org (all years)
	Fencing (foil)	628	637	
	Fencing (saber)	608	672	
	Table tennis	507	435	www.ittf.com (all years)
	Tennis	497	458	www.wtatennis.com (all years; females), www.atptour.com (all years; males)
	Squash	546	515	www.squashinfo.com (2007–2015), www.psaworldtour.com (2016)
Junior (study 2)	Fencing (épée)	984	1,131	www.fie.org (all years)
	Fencing (foil)	935	1,024	
	Fencing (saber)	923	1,035	
	Tennis	572	650	www.itftennis.com (all years; ITF junior rankings)

*In racket sports, all year-end rankings were obtained for end of December. In fencing, all rankings are season-end rankings and were assigned to the year a season ended for analyses (e.g., rankings for the season 2007/2008 were assigned to the year 2008)*.

### Data Analyses

In accordance with the primary study aim, data analyses were conducted separately by sex, sport, and discipline (fencing only). Inferential statistics were calculated on the frequencies of unique athletes (*N* = 6,784 in total) collapsed across the 10-year ranking period to prevent that the same individuals who were listed more than once in year-end world rankings were counted more than once. Indeed, across sports, the majority of athletes was listed twice or more among the top 200 year-end world rankings (57.3% in male saber to 79.9% in male tennis), with a small portion of athletes even listed in all year-end rankings (3.1% in male saber to 11.7% in male table tennis; see Supplementary Table 1 for a complete overview).

The reduced dataset on all unique athletes listed in the respective sports' rankings is also made available as Supplementary Material (see also Table 1).

To test for left-hander overrepresentation, the observed frequencies for left- and right-handedness were compared against expected normal population left-hander frequencies of 7.7% (females) and 10.3% (males) (Raymond et al., 1996), respectively, using chi-square goodness-of-fit tests. To test for RAE in birth distribution, athletes' birth months were first categorized into birth quartiles based on a universally assumed cutoff date of January 1st in all sports under consideration [i.e., quartile 1 (Q1) = January–March, Q2 = April–June, Q3 = July–September, Q4 = October–December]. In line with the traditional analytic approach in RAE research, chi-square goodness-of-fit tests were then conducted to compare observed frequencies against the assumption of uniform distribution. In addition, odds ratios (OR) were calculated based on the observed absolute frequencies for athletes born in Q1 relative to athletes born in Q4: OR = Q_1_ / Q_4_. Descriptively, OR > 1 indicates higher chances of athletes being born in Q1 than Q4, OR < 1 indicates the opposite, and OR = 1 reflects equal chances. The limits of the 95% confidence intervals (CI) on ORs were calculated as: exp[ln(OR) ± 1.96 * (sqrt((1/Q_1_) + (1/Q_4_)))], where exp = natural exponential function, ln = natural logarithm, and sqrt = square root.

Finally, 2 (handedness) × 4 (birth quartile) chi-square tests of independence were run to test whether birth distribution is skewed more heavily toward the “classical” RAE pattern in right- compared with left-handed athletes. In addition, ORs were also calculated based on the observed absolute frequencies as the ratio of right-handed athletes being born in Q1 as opposed to Q4 relative to left-handed athletes being born in Q1 as opposed to Q4: OR = (RH_Q1_/RH_Q4_)/(LH_Q1_/LH_Q4_). Thus, ORs reflect the chances of right-handers being born in the first vs. the fourth quartile relative to left-handers being born in the first vs. the fourth quartile. Descriptively, OR > 1 indicates higher chances in right- than left-handers, OR < 1 indicates the opposite, and OR = 1 reflects equal chances. The limits of the corresponding 95% CI were calculated as: exp[ln(OR) ± 1.96 ^*^ (sqrt((1/RH_Q1_) + (1/RH_Q4_) + (1/LH_Q1_) + (1/LH_Q4_)))].

For all inferential analyses, α was set at 0.05 and Cohen's (1988) *w* [i.e., sqrt(χ^2^/*N*)], where *N* = total number of cases considered in a particular analysis) was calculated as a standardized effect size measure for chi-square analyses. For each effect size *w*, the 90% confidence interval was calculated based on the noncentral chi-square files provided by Michael Smithson (http://www.michaelsmithson.online/stats/CIstuff/CI.html)^1^.

## Results and Discussion

### Handedness Distribution

In line with previous research (Raymond et al., 1996; Loffing, 2017), the number of left-handed athletes observed in the 10-year period was higher than expected from the normal population estimate, especially in the high time pressure sporting domains of foil (females: 22.77%, males: 25.75%), épée (females: 16.85%, males: 20.86%), and table tennis (females: 18.64%, males: 25.18%; Table 2). In these disciplines, effect size point estimates and *lower* limits of associated 90% CIs are located in Cohen's (1988) conventional area of medium (*w* = 0.3–0.5) to large effects (*w* ≥ 0.5), pointing toward a substantial advantage for left-handed athletes. Left-handedness was not more prevalent and thus likely not substantially beneficial to performance in squash (females: 6.77%, males: 8.97%) and tennis (females: 8.85%, males: 12.66%). In saber, an at least statistically significant overrepresentation was evident in female (11.68%) but not in male competition (12.50%), thus indicating no systematic left-hander overrepresentation in this discipline along with small effect sizes as opposed to the other two fencing disciplines.

**Table 2:** Absolute frequencies and results from chi-square goodness-of-fit tests related to handedness in senior athletes (study 1).

**Sex**	**Sport (discipline)**	**Handedness**					
		**L**	**R**	**N/A**	**χ^2^**	***p***	***w* and 90% CI**
Female	Fencing (épée)	106	523	0	74.13	<0.001	0.34 (0.28, 0.41)
	Fencing (foil)	143	485	0	200.69	<0.001	0.57 (0.5, 0.63)
	Fencing (saber)	71	537	0	13.54	<0.001	0.15 (0.08, 0.22)
	Table tennis	88	384	35	79.54	<0.001	0.41 (0.33, 0.49)
	Tennis	44	453	0	0.93	0.335	0.04 (0, 0.12)
	Squash	36	496	14	0.65	0.419	0.03 (0, 0.11)
Male	Fencing (épée)	136	516	0	78.68	<0.001	0.35 (0.28, 0.41)
	Fencing (foil)	164	473	0	164.48	<0.001	0.51 (0.44, 0.57)
	Fencing (saber)	84	588	0	3.52	0.061	0.07 (0, 0.14)
	Table tennis	107	318	10	101.80	<0.001	0.49 (0.41, 0.57)
	Tennis	58	400	0	2.77	0.096	0.08 (0, 0.15)
	Squash	46	467	2	0.99	0.321	0.04 (0, 0.12)

*L, left-handed; R, right-handed; N/A, handedness not available; w, standardized Cohen's (1988) effect size. df = 1 for all comparisons (see main text for details)*.

**Figure 1:**
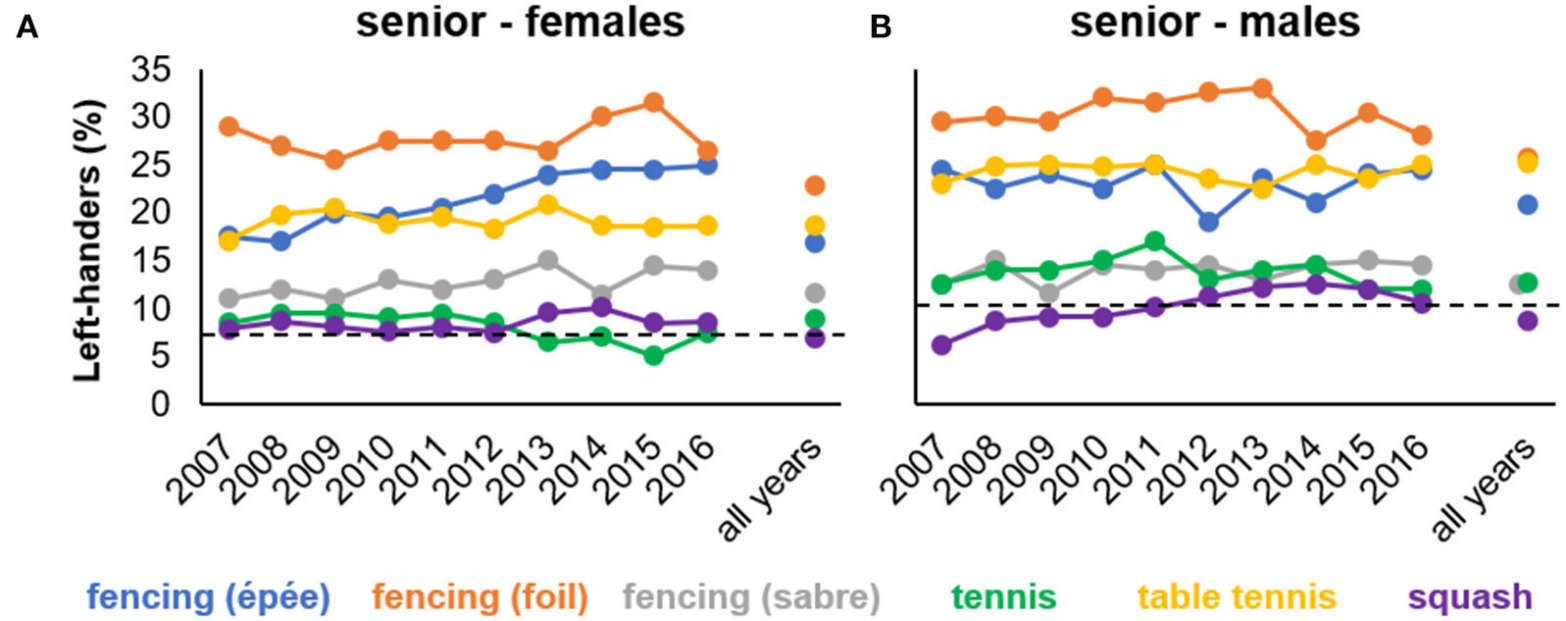
Percentages of left-handed athletes for each year-end ranking and collapsed across all years listed for a particular sport separately for **(A)** female and **(B)** male senior competition (study 1). Horizontal dashed lines represent reference values for normal population estimates of left-handedness in females (7.7%) and males (10.3%), respectively (Raymond et al., 1996).

In addition to the former findings on data collapsed across the 10-year ranking period, Figure 1 gives an overview of the temporal variability in year-end rankings' left-hander frequencies (cf. Goldstein and Young, 1996; Loffing et al., 2012; Loffing and Hagemann, 2015). Accordingly, in female and male competition, the proportion of left-handedness was relatively stable across years. Importantly, the temporal stability found here should not be interpreted as evidence for temporal stability of left-hander frequencies *per se*. Instead, stability might result from the relatively narrow 10-year window across which frequencies were considered and the large proportion of athletes listed twice or more among the top performers within that period (see Supplementary Table 1). Inspection of broader time windows, for example, in tennis (4 decades: Loffing et al., 2012), boxing (9 decades: Loffing and Hagemann, 2015), or baseball (11 decades: Goldstein and Young, 1996), revealed considerable temporal variation in left-hander frequencies.

### Birthdate Distribution

Irrespective of handedness, the “classical” RAE distributional pattern of birth frequencies was not systematically skewed toward quartiles 1 (January–March) and 2 (April–June) across sports. A RAE-like distribution of birth quartile frequencies was only indicated in female table tennis and saber as well as male tennis, with point estimates for Cohen's (1988) *w* and 90% CI *upper* limits below the conventional limit for a medium effect (Table 3). Likewise, odds ratios obtained from the comparison of frequencies observed for Q1 vs. Q4 provide indication for small RAE particularly in female table tennis (OR = 1.71) and male tennis (OR = 1.52; see Table 3). Given these findings, birth month does not seem to stand out as a prominent factor in explaining distributional differences among top-ranked senior athletes in the sports considered. This is in line with previous research on Olympic athletes (Baker et al., 2009).

**Table 3:** Absolute frequencies, results from chi-square goodness-of-fit tests, and odds ratios related to relative age in senior athletes (study 1).

**Sex**	**Sport (discipline)**	**Relative age**								
		**Q1**	**Q2**	**Q3**	**Q4**	**N/A**	**χ^2^**	***p***	***w* and 90% CI**	**OR and 95% CI**
Female	Fencing (épée)	166	170	166	127	0	7.83	0.050	0.11 (0, 0.17)	1.31 (1.04, 1.65)
	Fencing (foil)	180	165	143	140	0	6.87	0.076	0.10 (0, 0.16)	1.29 (1.03, 1.60)
	Fencing (saber)	182	140	145	141	0	7.99	0.046	0.11 (0.01, 0.17)	1.29 (1.04, 1.61)
	Table tennis	159	117	131	93	7	18.24	<0.001	0.19 (0.1, 0.26)	1.71 (1.32, 2.21)
	Tennis	143	132	115	107	0	6.40	0.094	0.11 (0, 0.17)	1.34 (1.04, 1.72)
	Squash	145	125	133	129	14	1.68	0.640	0.06 (0, 0.1)	1.12 (0.89, 1.42)
Male	Fencing (épée)	182	151	163	155	1	3.49	0.321	0.07 (0, 0.12)	1.17 (0.95, 1.45)
	Fencing (foil)	159	178	147	153	0	3.40	0.335	0.07 (0, 0.12)	1.04 (0.83, 1.30)
	Fencing (saber)	178	164	192	137	1	9.85	0.020	0.12 (0.04, 0.17)	1.3 (1.04, 1.62)
	Table tennis	122	100	113	97	3	3.76	0.289	0.09 (0, 0.15)	1.26 (0.96, 1.64)
	Tennis	128	133	113	84	0	12.72	0.005	0.17 (0.07, 0.23)	1.52 (1.16, 2.01)
	Squash	141	124	124	125	1	1.63	0.653	0.06 (0, 0.1)	1.13 (0.89, 1.44)

*Q1 … Q4, birth quartile 1 (January–March) … birth quartile 4 (October–December); N/A, birthdate not available; w, standardized Cohen's (1988) effect size; OR, odds ratio based on frequencies observed for Q1 and Q4 (see main text for details). df = 3 for all chi-square comparisons*.

Supplemental analyses revealed considerable variation in the year-wise relationship between birth month and birth frequency within and between sports in both male and female senior athletes (see Supplemental Material for details), indicating that the RAE phenomenon—be it present or not for a particular point in time—should not be assumed of constant magnitude across a broader time window (Schorer et al., 2020). This issue, however, might be of particular relevance at the senior level, where RAE have previously been demonstrated inconsistent (Schorer et al., 2020) and of smaller magnitude as opposed to the junior level (Cobley et al., 2009).

### Birthdate Distribution and Handedness

**Figure 2:**
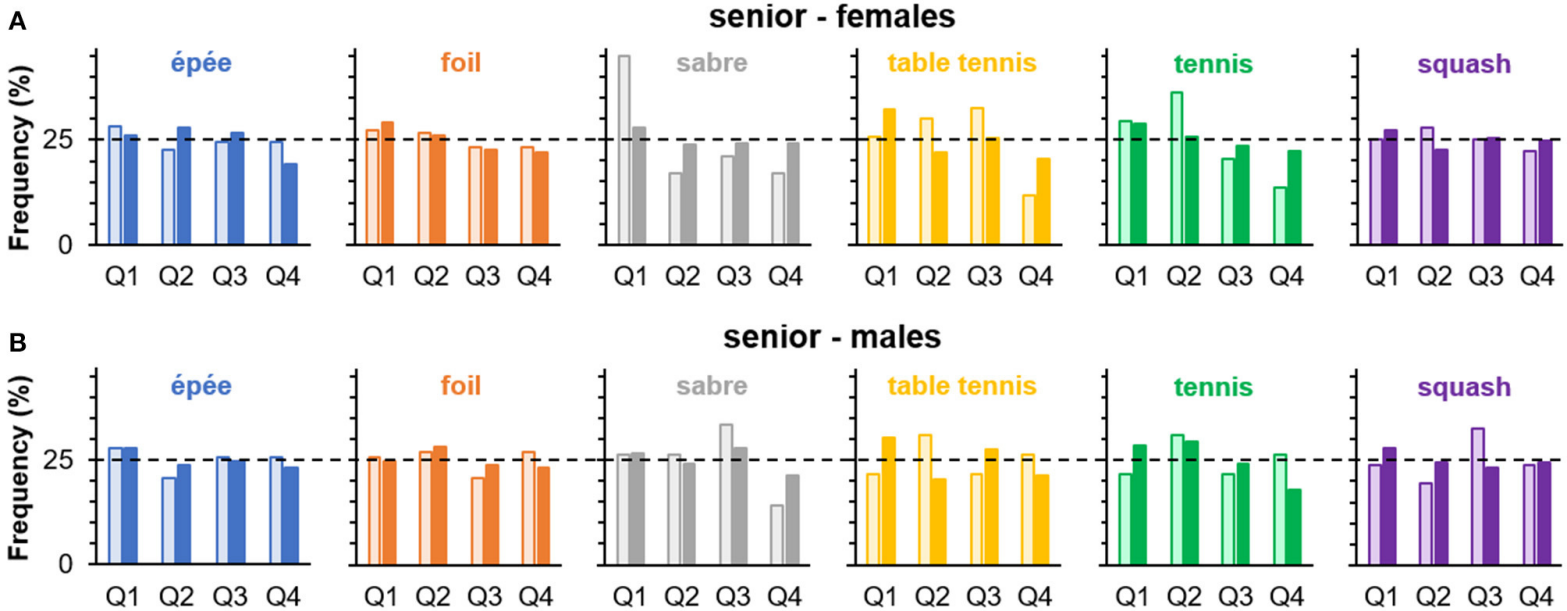
Percentages of birth quartile (Q1–Q4) frequencies in left- (□) and right-handers (■) separately for sport or discipline in **(A)** female and **(B)** male senior athletes (study 1). Horizontal dashed lines indicate expected frequencies under the assumption of a uniform distribution.

An overview of summary statistics relevant to the chi-square tests of independence between handedness and birth quartile is given in Table 4, and the corresponding relative frequencies are displayed in Figure 2. Across comparisons, effect size point estimates were mostly below Cohen's (1988) conventional threshold for small effects (*w* = 0.10), and the *upper* limits of the corresponding 90% CIs did not exceed the value of *w* = 0.20 in any comparison. Similarly, across comparisons, odds ratios oscillated around the value of 1 and there was no reliable indication of higher chances in right-handers being born in Q1 than Q4 compared with left-handers being born in Q1 than Q4 (Table 4). Overall, analyses do neither provide statistical support for the hypothesis that handedness and relative age are associated nor that the classical RAE pattern in birth quartile distribution is more pronounced in right- than left-handers. The association found in female saber fencers, for example, is even in contrast to the latter prediction as birth distribution was more skewed toward Q1 in left-handed [45.1%; *w* = 0.47, 90% CI (0.23, 0.64) for a within-handedness goodness-of-fit test against uniform distribution; OR_Q1:Q4_ = 2.67, 95% CI (1.37, 5.18)] than right-handed fencers [27.9%; *w* = 0.07, 90% CI (0, 0.12); OR_Q1:Q4_ = 1.16, 95% CI (0.92, 1.47)] (see Figure 2).

**Table 4:** Absolute frequencies, results from chi-square tests of independence between handedness and relative age, and odds ratios in senior athletes (study 1).

**Sex**	**Sport (discipline)**	**Left-handed**				**Right-handed**				**Chi^2^ test of independence**			
		**Q1**	**Q2**	**Q3**	**Q4**	**Q1**	**Q2**	**Q3**	**Q4**	**χ^2^**	***p***	***w* and 90% CI**	**OR and 95% CI**
Female	Fencing (épée)	30	24	26	26	136	146	140	101	2.44	0.486	0.06 (0, 0.11)	1.17 (0.65, 2.09)
	Fencing (foil)	39	38	33	33	141	127	110	107	0.19	0.979	0.02 (0, 0)	1.12 (0.66, 1.89)
	Fencing (saber)	32	12	15	12	150	128	130	129	9.08	0.028	0.12 (0.03, 0.18)	0.44 (0.22, 0.88)
	Table tennis	22	26	28	10	122	84	96	78	7.27	0.064	0.12 (0, 0.19)	0.71 (0.32, 1.58)
	Tennis	13	16	9	6	130	116	106	101	3.30	0.347	0.08 (0, 0.14)	0.59 (0.22, 1.62)
	Squash	9	10	9	8	133	111	124	121	0.52	0.914	0.03 (0, 0.05)	0.98 (0.37, 2.61)
Male	Fencing (épée)	38	28	35	35	144	123	128	120	0.80	0.848	0.04 (0, 0.06)	1.11 (0.66, 1.86)
	Fencing (foil)	42	44	34	44	117	134	113	109	1.39	0.708	0.05 (0, 0.09)	1.12 (0.68, 1.85)
	Fencing (saber)	22	22	28	12	156	142	164	125	2.64	0.451	0.06 (0, 0.11)	0.68 (0.32, 1.43)
	Table tennis	23	33	23	28	97	65	88	68	8.04	0.045	0.14 (0.01, 0.2)	1.74 (0.92, 3.27)
	Tennis	14	15	17	12	114	118	96	72	1.35	0.716	0.05 (0, 0.1)	1.36 (0.59, 3.10)
	Squash	11	9	15	11	130	114	108	114	2.22	0.529	0.07 (0, 0.12)	1.14 (0.48, 2.73)

*Q1 … Q4, birth quartile 1 (January–March) … birth quartile 4 (October–December); w, standardized Cohen's (1988) effect size; OR, odds ratio based on frequencies observed for right-handers in Q1 vs. Q4 relative to frequencies observed in left-handers in Q1 vs. Q4 (see main text for details). df = 3 for all chi-square comparisons*.

Supplemental analyses were conducted on a year-wise basis with the classification of left- and right-handed athletes as being born either in the first or second half of a year. Contrary to the analyses presented above on data collapsed across the 10-year-period, half-year classification was chosen for supplemental analyses due to otherwise low number of observations for left-handers' birth quartiles. The year-wise chances of right-handers being born in the first vs. the second half of a year relative to left-handers being born in the first vs. the second half of a year were relatively stable within each sport and rarely exceeded the values of 2 (higher chances in right-handers) and 0.5 (higher chances in left-handers; see online Supplemental Material for details). Collectively, results from the above analyses do not mirror previous reports on a lower prevalence of left-handed senior athletes born early in the year in comparison with their right-handed counterparts (Schorer et al., 2009; Loffing et al., 2010; Barrenetxea-Garcia et al., 2019).

## Study 2: Relative Age Effects and Handedness in World-Class Junior Athletes

In the former study, the detection of the “classical” RAE phenomenon and its hypothesized association with athletes' handedness might have been hindered by focusing on senior athletes. Remarkably, so far, research on a left-hander advantage in individual interactive sports has exclusively been reported on senior but not junior elite competition. There is compelling evidence that the RAE phenomenon as such reduces from junior to senior level, for example, likely due to the reduction in birth-month-related relative age differences with increasing age (Cobley et al., 2009). Investigating RAE in male- and female-talented Australian cricketers, Connor et al. (2019) suggested that birth distribution was skewed toward quartile 1 in both left- and right-handed batters and bowlers at the younger age groups (e.g., under 15, under 17), but no longer in left- and occasionally in right-handed players at older age groups (i.e., under 19 and state level). Consequently, provided that the RAE phenomenon is more pronounced and that left-handedness is also relevant to performance at the junior level, the hypothesized effect of athletes' handedness in conjunction with birth month might show through more clearly in that particular age group. In study 2, we put this prediction to test by investigating handedness and relative age in internationally top-ranked junior athletes in fencing and tennis.

## Materials and Methods

### Data Retrieval and Data Analyses

The steps related to data retrieval and data analyses were identical to study 1. Here, we focused on the sports of fencing and tennis. For fencing, as in study 1, data on the top 200 athletes were available for each year in each discipline. For tennis, the total number of players listed in year-end rankings ranged from 77 (2009 and 2011) to 98 (2014) in female players and the number of male players ranged from 86 (2009) to 101 (2012) per year-end ranking (see the dataset provided as Supplementary Material for details). Overall, a total of *N* = 7,254 unique junior athletes were included in the dataset (see Table 1 for an overview). Compared with senior athletes examined in study 1, the proportion of junior athletes listed twice or more among the top performers in year-end world rankings was clearly lower (36.7% in female tennis to 57.3% in female saber), with the maximum number of years ranging from 4 (male and female tennis) to 7 (all fencing disciplines in females and male foil; see Supplementary Table 1 for a complete overview). Of note, the athletes considered here as “juniors” are within the age group labeled as “adolescents” (15–18 years) in the meta-analytical review of Cobley et al. (2009).

## Results and Discussion

### Handedness Distribution

The number of left-handed junior athletes observed in the 10-year period was higher than expected from the normal population estimate especially in foil (females: 17.43%, males: 20.67%) and épée (junior females: 15.45%, junior males: 18.57%; Table 5). In these disciplines, effect size point estimates and the *lower* limits of the associated 90% CIs are located in Cohen's (1988) conventional area of medium effects, suggesting a marked left-hander advantage. For tennis (females: 9.27%, males: 15.3%) and saber (females: 9.43%, males: 11.3%), the results indicate no systematic or marked—in terms of effect sizes and related confidence intervals—left-hander overrepresentation in these sports. Only in male tennis players, there is a small significant effect with an overrepresentation of left-handers.

**Table 5:** Absolute frequencies and results from chi-square goodness-of-fit tests related to handedness in junior athletes (study 2).

**Sex**	**Sport (discipline)**	**Handedness**					
		**L**	**R**	**N/A**	**χ^2^**	***p***	***w* and 90% CI**
Female	Fencing (épée)	152	832	0	83.10	<0.001	0.29 (0.24, 0.34)
	Fencing (foil)	163	772	0	124.63	<0.001	0.37 (0.31, 0.42)
	Fencing (saber)	87	836	0	3.87	0.049	0.06 (0, 0.12)
	Tennis	53	519	0	1.97	0.160	0.06 (0, 0.13)
Male	Fencing (épée)	210	921	0	83.68	<0.001	0.27 (0.22, 0.32)
	Fencing (foil)	211	810	3	118.75	<0.001	0.34 (0.29, 0.39)
	Fencing (saber)	117	918	0	1.13	0.288	0.03 (0, 0.08)
	Tennis	99	548	3	17.52	<0.001	0.16 (0.1, 0.23)

*L, left-handed; R, right-handed; N/A, handedness not available; w, standardized Cohen's (1988) effect size. df = 1 for all comparisons (see main text for details)*.

**Figure 3:**
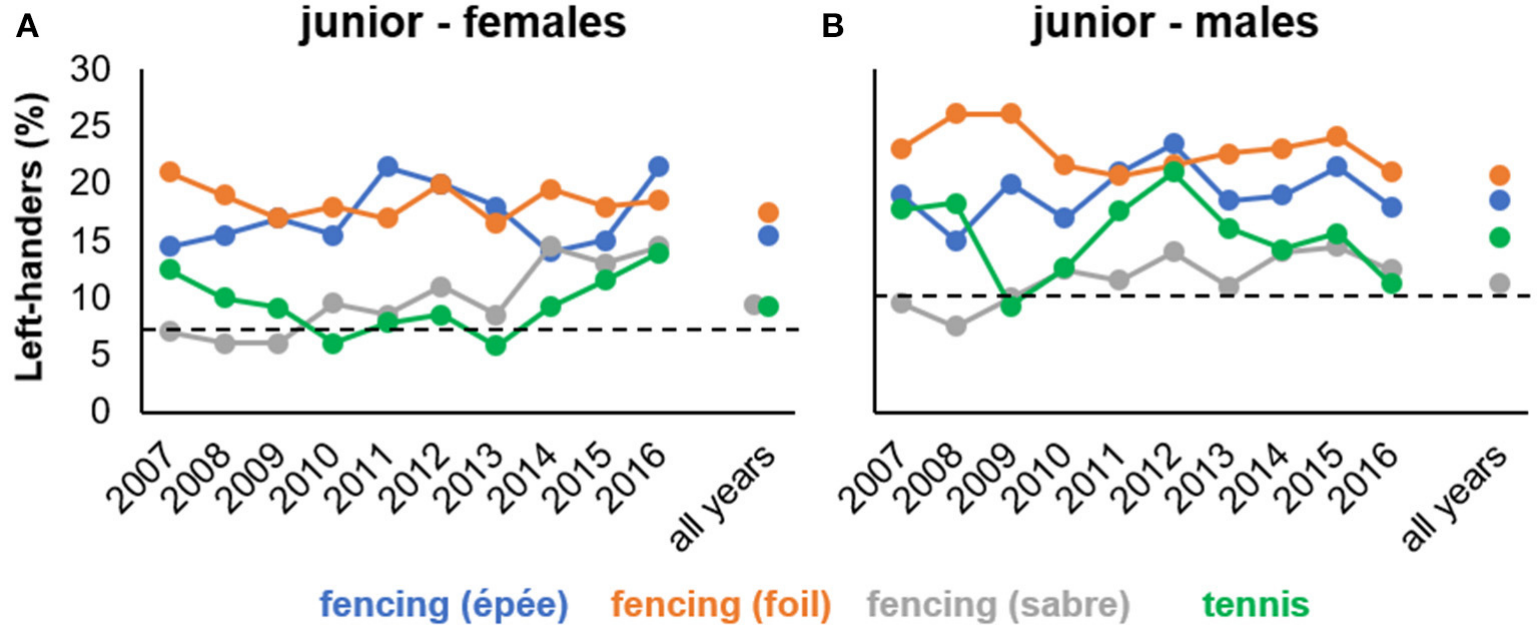
Percentages of left-handed athletes for each year-end ranking and collapsed across all years listed for a particular sport separately for **(A)** female and **(B)** male junior competition (study 2). Horizontal dashed lines represent reference values for normal population estimates of left-handedness in females (7.7%) and males (10.3%), respectively (Raymond et al., 1996).

As illustrated in Figure 3, across the 10-year period, left-hander frequencies were quite variable ranging, for example, from 6% (2008 and 2009) to 14.5% (2014) in female saber or from 9.3% (2009) to 21% (2012) in male tennis. This finding contrasts with the stable temporal patterns found in senior athletes (study 1) and highlights that snapshots of left-hander frequencies for a particular year may give an unreliable estimate of the potential role of handedness in junior competition. We speculate that the stronger temporal variation in left-hander frequencies could be due to larger fluctuation of athletes entering and leaving top-ranking positions in juniors compared with seniors. The latter is reflected in larger total numbers of junior than senior athletes included in our fencing and tennis samples (see Table 1) as well as in the lower proportions of junior compared with senior athletes listed twice or more in year-end world rankings (see Supplementary Table 1). Top juniors move up to the seniors quickly, once they are ready, while seniors may remain for several years up to more than a decade in the same competitive system.

### Birthdate Distribution

The “classical” RAE distributional pattern of birth frequencies being skewed toward quartiles 1 (January–March) and 2 (April–June) was evident in both female and male junior athletes (except for female saber; see Table 6). The corresponding effect size point estimates, however, were located in the lower half of Cohen's (1988) conventional region of small effects (*w* = 0.1 to 0.3), and the *upper* limits of the associated 90% CIs often did not exceed the value of *w* = 0.2. A notable exception is male tennis, where a medium-sized effect associated with a gradual decrease of birth frequencies from Q1 to Q4 was found (cf. Edgar and O'Donoghue, 2005). Similarly, odds ratios obtained from the comparison of frequencies observed for Q1 vs. Q4 provide indication for a RAE, particularly in male (OR = 2.18) and female tennis (OR = 1.75; see Table 6).

**Table 6:** Absolute frequencies, results from chi-square goodness-of-fit tests, and odds ratios related to relative age in junior athletes (study 2).

**Sex**	**Sport (discipline)**	**Relative age**								
		**Q1**	**Q2**	**Q3**	**Q4**	**N/A**	**χ^2^**	***p***	***w* and 90% CI**	**OR and 95% CI**
Female	Fencing (épée)	276	289	233	186	0	26.50	<0.001	0.16 (0.1, 0.21)	1.48 (1.23, 1.79)
	Fencing (foil)	270	251	223	191	0	15.21	0.002	0.13 (0.06, 0.17)	1.41 (1.17, 1.70)
	Fencing (saber)	255	220	236	212	0	4.69	0.196	0.07 (0, 0.11)	1.20 (1.00, 1.44)
	Tennis	175	146	151	100	0	20.60	<0.001	0.19 (0.11, 0.25)	1.75 (1.37, 2.24)
Male	Fencing (épée)	321	296	282	232	0	14.91	0.002	0.11 (0.05, 0.16)	1.38 (1.17, 1.64)
	Fencing (foil)	281	295	231	217	0	16.77	0.001	0.13 (0.07, 0.17)	1.29 (1.08, 1.55)
	Fencing (saber)	287	273	261	214	0	11.63	0.009	0.11 (0.04, 0.15)	1.34 (1.12, 1.60)
	Tennis	227	182	137	104	0	53.00	<0.001	0.29 (0.21, 0.35)	2.18 (1.73, 2.75)

*Q1 … Q4, birth quartile 1 (January–March) … birth quartile 4 (October–December); N/A, birthdate not available; w, standardized Cohen's (1988) effect size; OR, odds ratio based on frequencies observed for Q1 and Q4 (see main text for details). df = 3 for all chi-square comparisons*.

Supplemental analyses revealed variation in the year-wise relationship between birth month and birth frequency within and between sports in both male and female athletes (see Supplemental Material), again supporting the notion that the RAE is not necessarily a temporally stable phenomenon even within the same sport or discipline. Compared with the finding for senior athletes in study 1, however, the linear relationships revealed for junior athletes appear more stable and consistent with the “classical” RAE phenomenon known from the literature (Cobley et al., 2009).

### Birthdate Distribution and Handedness

**Figure 4:**
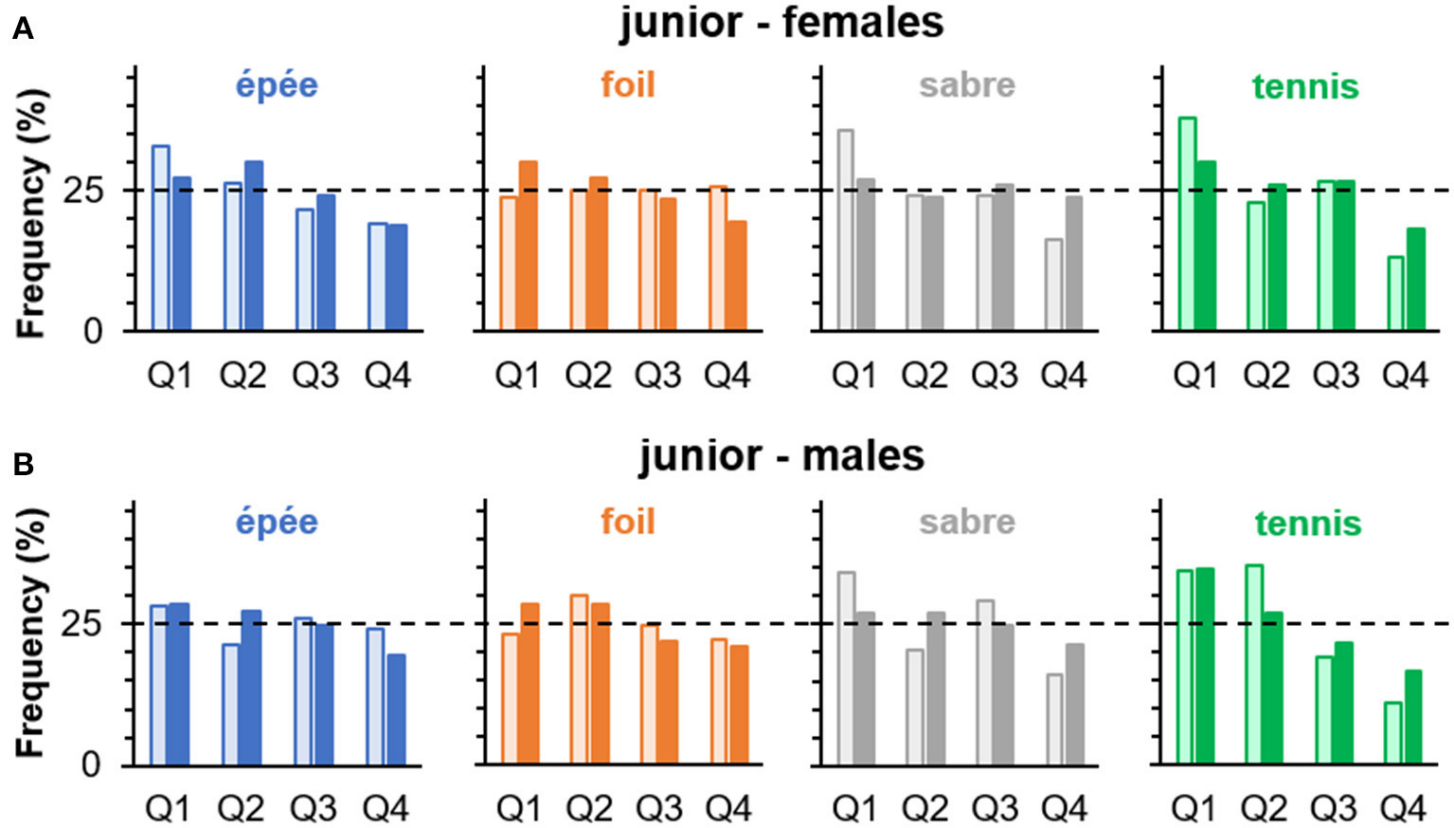
Percentages of birth quartile (Q1–Q4) frequencies in left- (□) and right-handers (■) separately for sport or discipline in **(A)** female and **(B)** male junior athletes (study 2). Horizontal dashed lines indicate expected frequencies under the assumption of a uniform distribution.

Table 7 gives an overview of the summary statistics relevant to the chi-square tests of independence between handedness and birth quartile, and the corresponding relative frequencies are illustrated in Figure 4. For all comparisons, effect size point estimates were below Cohen's (1988) conventional threshold for small effects (*w* = 0.10), and the *upper* limits of the corresponding 90% CIs rarely exceed that particular threshold. With regard to odds ratios, there was no systematic and reliable indication of higher chances in right-handers being born in Q1 than Q4 compared with left-handers being born in Q1 than Q4 (Table 7). If at all, there was a trend for such pattern in female foil; however, the opposite was found in female saber. Overall, the analyses do neither provide convincing statistical support for the hypothesis that handedness and relative age are interrelated in fencing or tennis nor that the classical RAE pattern in birth quartile distribution is more pronounced in right- than left-handed athletes.

**Table 7:** Absolute frequencies, results from chi-square tests of independence between handedness and relative age, and odds ratios in junior athletes (study 2).

**Sex**	**Sport (discipline)**	**Left-handed**				**Right-handed**				**Chi^2^-test of independence**			
		**Q1**	**Q2**	**Q3**	**Q4**	**Q1**	**Q2**	**Q3**	**Q4**	**χ^2^**	***p***	***w* and 90% CI**	**OR and 95% CI**
Female	Fencing (épée)	50	40	33	29	226	249	200	157	2.37	0.499	0.05 (0, 0.09)	0.83 (0.51, 1.38)
	Fencing (foil)	39	41	41	42	231	210	182	149	4.78	0.189	0.07 (0, 0.11)	1.67 (1.03, 2.70)
	Fencing (saber)	31	21	21	14	224	199	215	198	4.29	0.232	0.07 (0, 0.11)	0.51 (0.26, 0.99)
	Tennis	20	12	14	7	155	134	137	93	1.77	0.620	0.06 (0, 0.1)	0.58 (0.24, 1.43)
Male	Fencing (épée)	59	45	55	51	262	251	227	181	4.18	0.243	0.06 (0, 0.1)	1.25 (0.82, 1.90)
	Fencing (foil)	49	63	52	47	231	231	178	170	2.48	0.480	0.05 (0, 0.09)	1.30 (0.83, 2.04)
	Fencing (saber)	40	24	34	19	247	249	227	195	5.73	0.125	0.07 (0, 0.11)	0.60 (0.34, 1.07)
	Tennis	34	35	19	11	191	147	118	92	4.09	0.252	0.08 (0, 0.13)	0.67 (0.33, 1.39)

*Q1 … Q4, birth quartile 1 (January–March) … birth quartile 4 (October–December); w, standardized Cohen's (1988) effect size; OR, odds ratio based on frequencies observed for right-handers in Q1 vs. Q4 relative to frequencies observed in left-handers in Q1 vs. Q4 (see main text for details). df = 3 for all chi-square comparisons*.

Supplemental analyses revealed that the year-wise chances of right-handers being born in the first vs. the second half of a year relative to left-handers being born in the first vs. the second half of a year were relatively stable within each sport and rarely exceeded the values of 2 (higher chances in right-handers) and 0.5 (higher chances in left-handers; see online Supplemental Material for details). Collectively, similar to study 1 for senior athletes, the present findings for junior athletes do not match with previous reports on the predominance of the “classical” RAE phenomenon in right- as opposed to left-handed athletes (Schorer et al., 2009; Loffing et al., 2010; Barrenetxea-Garcia et al., 2019; Connor et al., 2019).

## General Discussion

Here, in two studies, we tested whether top-ranked male and female athletes' left-handedness is associated with a lower likelihood of suffering the commonly observed RAE phenomenon as compared with right-handedness in international elite interactive individual sports. Study 1 included senior athletes in fencing (épée, foil, and saber), table tennis, tennis, and squash, while study 2 focused on junior athletes in fencing (all disciplines) and tennis.

In line with previous research on handedness in interactive sports, left-handers were clearly overrepresented in some but not all sports considered (Loffing and Hagemann, 2016; Fagan et al., 2019). At the senior level, an overrepresentation was particularly evident in foil, épée, and table tennis in both female and male competitions (Raymond et al., 1996; Loffing, 2017), whereas at the junior level, this was most evident in foil and épée (females and males) but also in male tennis. The finding of a left-hander overrepresentation at the junior level, which has vastly been neglected in previous research on a handedness-related performance advantage so far (Schorer et al., 2016), provides new insight into the role handedness may play for performance and career development in these different sports. Albeit direct comparisons between the senior and junior findings may be limited, one tentative interpretation of the tennis data is that handedness might still be performance relevant at the junior level in male competition, but no longer stands out at the senior level. The mechanisms behind obviously cannot be clearly identified from the present study design. However, we speculate that this could be due, for example, to better opportunities for targeted match preparation against left-handed senior opponents and more flexible expertise-related adaptation to unfamiliar playing conditions (i.e., playing against a rare left- rather than a common right-hander) (Loffing et al., 2012) in conjunction with relatively lower spatiotemporal pressure compared with other high-pressure interactive sports where a left-hander advantage persists at the senior level (e.g., table tennis; Loffing, 2017). In the fast-interactive fencing disciplines of foil and épée, the left-hander advantage clearly identifiable at the junior level in both males and females persists at the senior level, even to a stronger degree in terms of standardized effect sizes (cf. Tables 2, 4). The lack of consistently enhanced left-hander frequencies in saber competition is surprising in this respect, but on the other hand, this fits with previous research on handedness in fencing (Azémar et al., 1983; Raymond et al., 1996). One explanation for this counterintuitive finding is that the fencing disciplines may impose different demands on sensorimotor processing, favoring either the right hemisphere and thus left-hand control (épée and foil) or the left hemisphere and thus right-hand control (saber) (Boulinguez, 1999; Boulinguez et al., 2001). Since based on the present data we can neither confirm that proposition nor exclude alternative explanations such as greater fighting distance in saber than épée and foil (Raymond et al., 1996), we refrain from speculating further about the potential underlying mechanisms and instead assign this as homework for more in-depth laterality research in fencing. Collectively, in light of the above considerations, it appears to have both theoretical importance and practical relevance to determine the potentially facilitating role laterality, and left-handedness in particular, may play during athletes' developmental pathway from junior to elite international senior competition in interactive sports (Schorer et al., 2016; Connor et al., 2019, 2020).

In general, RAE were within the previously reported range from small to null effect sizes for world-class athletes (Baker et al., 2009). *Post hoc* power estimation using G*Power (version 3.1.9.6; Faul et al., 2007) revealed that the minimum subgroup sample size observed in male senior table tennis (*N* = 435; see Table 1) allowed the detection of an effect of Cohen's (1988) *w* = 0.2 or more extreme using goodness-of-fit tests, provided that it truly exists, given α = 0.05 and *df* = 3 with a power of 95.3%. Thus, sample sizes were reasonably large enough to detect a small to medium RAE using Cohen's (1988) tentative effect size terminology with sufficiently large power. The *post hoc* power for detecting an even smaller effect of at least *w* = 0.1 with all else being equal, however, ranged from 38.8% (male senior table tennis) to 81.6% (male junior épée, *N* = 1,131). Consequently, conclusive interpretation of such small or even smaller effects is limited. This needs to be kept in mind in the following paragraph where we contemplate our RAE findings as well as in general by those who consider the effects of such magnitude as practically relevant.

In our samples, the RAE identified were only larger for junior athletes in tennis in comparison with senior athletes, while in all fencing disciplines, they were highly similar. The results for tennis are in line with the previous observations that RAE decrease from the age group of 15–18-year olds to seniors (Cobley et al., 2009). The contrast to the missing clear age differences in all fencing disciplines might be best explained by the competition hypothesis (Schorer et al., 2009; Lemez et al., 2014; Wattie et al., 2015). There is presumably lower competition in fencing around the world, as there are much less athletes in this sport than for example in tennis, which would be predicted to result in larger effects in tennis than in fencing. Comparing the RAE between sexes shows that for most sports the effect sizes do only marginally differ. In junior tennis, the effect is close to a medium level in male players, according to the effect size conventions by Cohen (1988), whereas the effect (point estimate and confidence interval) is within the range of a small effect in female players. The latter is in line with previous research revealing larger effects for male than female athletes (Cobley et al., 2009; Smith et al., 2018). Surprisingly, there seems to be an opposite trend in senior table tennis. Previous research on table tennis demonstrated larger effects for males than for females in youth players (Faber et al., 2020a,b). Future investigations would need to check if the varying effects found across studies might be due to country-specific influences (e.g., differences in talent identification and selection programs) or if other constraints can be identified that interact here. Taken together, the present results suggest that individual constraints like sex differ only marginally in their association with RAE in the individual interactive sports considered here at both the junior and senior levels.

Considering the hypothesized association between RAE and handedness, the present research does not provide empirical support for the assumption that left-handedness is associated with lower likelihood of suffering the commonly observed RAE phenomenon as compared with right-handedness. In this respect, the comprehensive analyses provided here are an important addition to the paucity of evidence on such hypothesized association in individual interactive sports (Loffing et al., 2010). We speculate that the failure to detect such association, provided that it truly exists at all, might be due to our focus on international elite samples, for which only small to null RAE were identified. Furthermore, given that handedness obviously is a performance-relevant trait in some of the sports investigated here (i.e., épée, foil, and table tennis in particular), the competition for limited resources to develop further and move up the sporting career ladder may be similarly severe for both left- and right-handers in these sports, thus resulting in similar selective pressure acting upon them and making both handedness groups almost equally prone to possible birth-related inequalities (Baker et al., 2009; Cobley et al., 2009; Schorer et al., 2020). Since in the present study our focus was on international competition, future work may need to check whether the pattern of null findings reported here also holds at the national level. Furthermore, it is vital to keep in mind that handedness might still interact with RAE in other areas like team sports, where positional demands like in handball and water polo (Schorer et al., 2009; Barrenetxea-Garcia et al., 2019) or strategic considerations to enhance team flexibility as suggested in batting sports (Brooks et al., 2004; Hirotsu and Wright, 2005) may favor the selection of left-handed players irrespective of their relative age (Connor et al., 2019). A similar principle might also apply to the selection of rare left-footed players, for example, in soccer (Verbeek et al., 2017). Future research might want to focus on younger age groups during their development with expected larger RAE. The athletes of the current study have been through all developmental systems and made it to the top. World-class athletes, however, might not be substantially affected by the developmental factor relative age anymore (Baker et al., 2009; Cobley et al., 2009; Schorer et al., 2020). The present findings add to the developmental systems model (Wattie et al., 2015) by highlighting that handedness may indeed be a performance-relevant trait, which, however, was not found to substantially interact with RAE in the elite interactive individual sports considered here. Future consideration of the abovementioned steps like focusing on a sport's national level as well as extending the view on team sports is suggested to further unravel the potential interaction between laterality and RAE as proposed by the model.

One potential methodological limitation is that we were unable to check for cutoff dates in all countries included in the analyses. Given that the IOC ruled out the first of January as international cutoff date, however, this might be more of a theoretical rather than a serious “results-biasing” problem. Still, the UK is known as an exception in applying September 1st as the cutoff date for national level youth competition (Till et al., 2010; Kelly et al., 2020a,b). To account for this, we rerun all analyses reported for studies 1 and 2 with UK athletes' birthdates being classified relative to September 1st and birthdates of athletes from all other nations relative to January 1st. The corresponding results are reported in detail in Section B of the Supplementary Material. In essence, the results obtained from the “mixed cutoff date” classification do not markedly differ from the results obtained from the uniform cutoff date (i.e., January 1st) reported here in the main text. Consequently, as we are not aware of any other (big) nation employing a cutoff date different to January 1st at the national level, we are confident that the findings reported here are reliable and not confounded or biased by insufficiently defined cutoff date criteria. The use of an estimated uniform distribution for the goodness-of-fit tests on birthdate distributions might constitute another limitation as recently outlined by Delorme and Champely (2015). We therefore considered these authors' recommendation and ran additional analyses against a day-corrected distribution of expected birth quartile frequencies. Importantly, the effect size point estimates as well as the limits of the corresponding confidence intervals are almost identical to those obtained from the analyses reported here in Tables 2, 4 for a uniform distribution assumption (see Section A of the Supplementary Material for details). Thus, the above-discussed conclusions on RAE remain the same irrespective of whether the inferential results from uniform or day-corrected tests are considered. As another final limitation, sample sizes were restricted through our focus on the top performers in the respective sports. Firm conclusions regarding a potential association between RAE and handedness are limited by the small samples of left- compared with right-handed athletes, particularly in the domains of squash and tennis.

Taken together, the two studies reported here sought to close a gap in research on the association of RAE and handedness. Given that our sample comprised world-class athletes, our results apply to the association of handedness and RAE at the final stages of the development toward sporting expertise. Recently, the developmental importance of RAE has been emphasized again (Schorer et al., 2020). Therefore, future research on laterality and RAE is recommended to first look at younger age groups, when athletes are in their main developmental period, and second, research would highly benefit from more longitudinal data. Then, dropouts from, joiners in, and remainders in the system could be identified, which would allow a much closer look at the hypothesized association between handedness and RAE.

## Data Availability Statement

The original contributions presented in the study are included in the article/Supplementary Material, further inquiries can be directed to the corresponding author/s.

## Ethics Statement

Ethical review and approval was not required for the study on human participants in accordance with the local legislation and institutional requirements. Written informed consent from the participants' legal guardian/next of kin was not required to participate in this study in accordance with the national legislation and the institutional requirements. Written informed consent was not obtained from the individual(s), nor the minor(s)' legal guardian/next of kin, for the publication of any potentially identifiable images or data included in this article.

## Author Contributions

Both authors developed the study concept and contributed to the study design. Testing and data collection were performed under the supervision of FL. FL performed the data analysis. FL and JS interpreted the data. FL and JS drafted the manuscript. Both authors approved the final version of the manuscript for submission.

## Conflict of Interest

The authors declare that the research was conducted in the absence of any commercial or financial relationships that could be construed as a potential conflict of interest.

## Footnotes

^1^The SPSS-script provided by Michael Smithson allows calculation of the confidence intervals on the noncentrality parameter lambda (λ_low_, λ_upp_) of the noncentral chi-square distribution. Based on these calculations, the lower (*w*_low_) and upper (*w*_upp_) bounds of the confidence interval on Cohen's *w* are obtained *via w*_low_ = sqrt[λ_low_/*N*] and *w*_upp_ = sqrt[λ_upp_/*N*], respectively.
