# Supplementary material for: Handedness and Relative Age in International Elite Interactive Individual Sports Revisited
Source: Front Sports Act Living. 2021 Mar 31;3:662203. doi: 10.3389/fspor.2021.662203 (PMC8044324; doi:10.3389/fspor.2021.662203)
Supplement: Supplementary file 1 [file Data_Sheet_1.PDF]

Supplementary Material

Supplementary Table 1. Absolute frequencies on the number of years unique athletes were listed among the top performers in senior and junior year-end world rankings between 2007 and 2016 (see main text for details).

			**Number of Years**									
**Age**	**Sex**	**Sport (Discipline)**	*1*	*2*	*3*	*4*	*5*	*6*	*7*	*8*	*9*	*10*
senior	female	fencing (épée)	228	126	77	39	36	29	37	18	15	24
		fencing (foil)	232	119	70	48	38	37	23	19	17	25
		fencing (sabre)	209	125	65	50	38	31	23	24	13	30
		table tennis	130	80	72	49	31	36	35	14	30	30
		tennis	123	89	52	47	41	30	39	21	21	34
		squash	145	108	79	50	40	29	21	18	23	33
	male	fencing (épée)	259	109	79	62	34	30	17	20	11	31
		fencing (foil)	248	121	63	46	36	36	23	21	18	25
		fencing (sabre)	287	117	67	49	35	35	22	18	21	21
		table tennis	94	57	61	29	30	31	36	19	27	51
		tennis	92	75	64	37	35	40	27	22	22	44
		squash	139	90	55	54	39	39	22	17	19	41
junior	female	fencing (épée)	458	238	146	95	35	11	1			
		fencing (foil)	406	229	150	87	42	19	2			
		fencing (sabre)	394	218	151	96	52	11	1			
		tennis	362	167	39	4						
	male	fencing (épée)	589	302	171	54	13	2				
		fencing (foil)	502	233	171	76	38	3	1			
		fencing (sabre)	488	272	163	86	21	5				
		tennis	404	218	27	1						

In the following sections, we provide the results of additional analyses conducted to supplement the analytic procedure and results reported in the main text. For ease of reading, we will report the respective results combined for both senior (Study 1) and junior athletes (Study 2). Moreover, the following sections are split into two parts. In section A, the same cut-off date of January 1st is applied to all athletes irrespective of their nationality as is also reported in the main text. In section B, September 1st is used as cut-off date for all athletes from the UK (this is the cut-off date used at the UK national level in academic and sport settings; Till et al., 2010; Kelly et al., 2020a; Kelly et al., 2020b) in combination with January 1st for athletes from all other nations. We were not aware of any other nations apart from the UK to use nation-specific cut-off dates that deviate from cut-off dates at the international level.

SECTION A: UNIFORM CUT-OFF DATE AT JANUARY 1ST FOR ATHLETES FROM ALL NATIONS (as reported in the main text)

Day-corrected comparisons of birth quartile distributions

The chi-square goodness-of-fit tests for birth date distributions reported in the main text were conducted under the assumption of a uniform probability distribution in the respective reference populations. That approach, however, was recently criticized for e.g. being prone to resulting in a biased chi-square statistic (Delorme and Champely, 2015). To account for that argument, here we additionally report the results from day-corrected chi-square goodness-of-fit tests on birth quartile distribution as recommended by Delorme and Champely (2015), with expected relative frequencies for quartiles 1 to 4 as follows: $Q1_{exp}$ = 24.71%, $Q2_{exp}$ = 24.91%, $Q3_{exp}$ = $Q4_{exp}$ = 25.19%.

The results obtained from the day-corrected tests are summarized in Supplementary Table 2. A comparison with the results reported in the main text for the uniform distribution assumption reveals marginal differences only. As could have been expected in advance due to only slight differences in expected proportions under a uniform vs. day-corrected distribution, effect size point estimates and the corresponding 90% confidence interval limits are almost identical. Unlike the results reported in the main text, for day-corrected tests the frequency distributions observed in senior female épée and foil are now statistically significant different from the expected distribution, while was not the case for the test against a uniform distribution. Importantly, however, the size of the effects as well as the precision of the effect size estimation do not markedly differ from those obtained for the uniform distribution assumption (see Supplementary Table 2).

Supplementary Table 2. Results from chi² goodness-of-fit tests (vs. day-corrected distribution) related to relative age in senior and junior athletes plus effect size and 90% CI obtained from the uniform distribution tests (see main text for details) to facilitate comparison between test results.

			**day-corrected**			**uniform**
**Age**	**Sex**	**Sport (Discipline)**	χ^2	*p*	*w* & 90% CI	*w* & 90% CI
senior	female	fencing (épée)	8.44	.038	0.12 [0.02, 0.17]	0.11 [0, 0.17]
		fencing (foil)	7.99	.046	0.11 [0.01, 0.17]	0.10 [0, 0.16]
		fencing (sabre)	8.98	.030	0.12 [0.03, 0.18]	0.11 [0.01, 0.17]
		table tennis	19.45	< .001	0.2 [0.11, 0.26]	0.19 [0.1, 0.26]
		tennis	7.33	.062	0.12 [0, 0.18]	0.11 [0, 0.17]
		squash	2.02	.568	0.06 [0, 0.11]	0.06 [0, 0.1]
	male	fencing (épée)	4.05	.256	0.08 [0, 0.13]	0.07 [0, 0.12]
		fencing (foil)	3.84	.280	0.08 [0, 0.13]	0.07 [0, 0.12]
		fencing (sabre)	10.15	.017	0.12 [0.04, 0.18]	0.12 [0.04, 0.17]
		table tennis	4.17	.244	0.1 [0, 0.16]	0.09 [0, 0.15]
		tennis	13.65	.003	0.17 [0.08, 0.24]	0.17 [0.07, 0.23]
		squash	2.06	.561	0.06 [0, 0.11]	0.06 [0, 0.1]
junior	female	fencing (épée)	28.61	< .001	0.17 [0.11, 0.22]	0.16 [0.1, 0.21]
		fencing (foil)	17.05	.001	0.14 [0.07, 0.18]	0.13 [0.06, 0.17]
		fencing (sabre)	5.47	.140	0.08 [0, 0.12]	0.07 [0, 0.11]
		tennis	21.92	< .001	0.2 [0.12, 0.26]	0.19 [0.11, 0.25]
	male	fencing (épée)	16.74	.001	0.12 [0.06, 0.16]	0.11 [0.05, 0.16]
		fencing (foil)	18.63	< .001	0.13 [0.07, 0.18]	0.13 [0.07, 0.17]
		fencing (sabre)	13.07	.004	0.11 [0.05, 0.16]	0.11 [0.04, 0.15]
		tennis	56.08	< .001	0.29 [0.22, 0.35]	0.29 [0.21, 0.35]

Note: *w* = standardized Cohen's (1988) effect size calculated as sqrt(χ^2 / *N*) with *N* = total number of observations; df = 3 for all comparisons.

Temporal stability of the association between birth month and birth frequency

Since collapsing data across a 10-year period hides possible temporal variability in the association between birth month and birth frequency (Schorer et al., 2020) as well as to account for the potential loss of information when categorizing birth months into quartiles (Loffing, 2016), birth quartile analyses reported in the main text were supplemented with year-wise and collapsed data on non-parametric linear relationships (Spearman's rho) between birth month and frequency of birth. Under the condition of an 'optimal' or 'classical' RAE distribution, birth frequencies should gradually decrease from January (coded as 1) to December (coded as 12), resulting in a negative value for Spearman's rho.

Supplementary Figure 1 reveals 'the dance of Spearman's rho', indicating considerable variation in the relationship between birth month and birth frequency within and between sports/disciplines in both males and females age groups. Overall and tentatively in line with the frequency analyses on collapsed

data reported in the main text, the linear relationships appear more stable and consistent with RAE in junior (Supplementary Figure 1B) than senior competition (Supplementary Figure 1A).

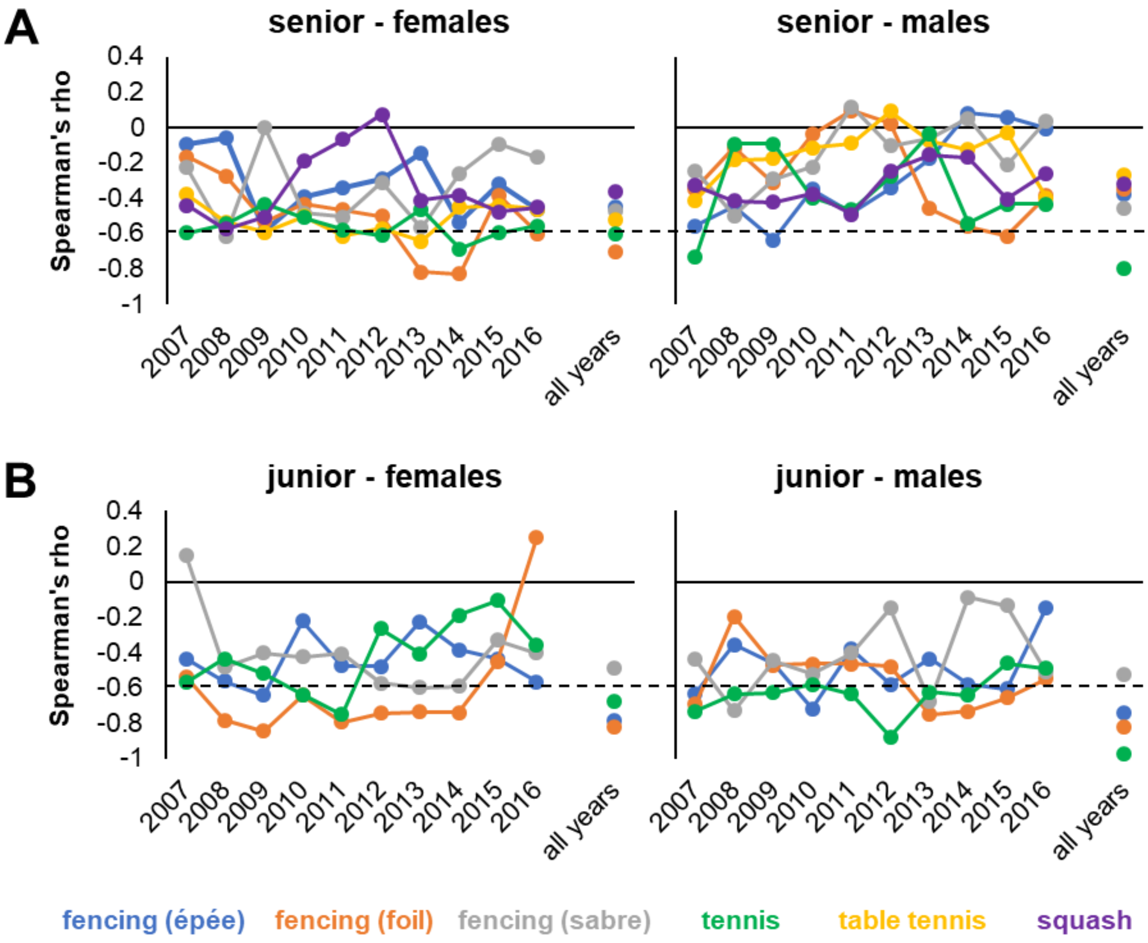

Supplementary Figure 1. Year-wise association between birth month and frequency of birth (Spearman's rho) for a particular sport separately for (A) senior and (B) junior competition. Horizontal dashed lines indicate the critical value for Spearman's rho (±0.587) for $N = 12$, $\alpha = .05$ (two-tailed).

Temporal stability of the association between handedness and time of birth

To reveal potential temporal variation in the association between handedness and birth quartile, data were additionally examined on a year-wise basis using a descriptive, graphical inspection of odds ratios (OR). Specifically, OR were calculated to reflect the chances of right-handed athletes being born in the first vs. second half of the year relative to left-handed athletes being born in the first vs. second half of a year. Accordingly, OR values larger (smaller) than 1 indicate higher (lower) chances in right-handed as compared to left-handed athletes, while a value of 1 means that chances are equal in both handedness groups.

Year-wise odds ratios are shown in Supplementary Figure 2. Overall, except for squash in senior males 2015 (OR = 3.95) and 2016 (OR = 2.71; Supplementary Figure 2A) as well as tennis in junior females

2013 (OR = 5.53; Supplementary Figure 2B) odds ratios were relatively stable within sports across years. On rare occasions, OR values exceeded the value of 2 (senior females: 1, senior males: 5, junior females: 1, junior males: 1) or fell below the value of 0.5 (senior females: 7 [tennis only], senior males: 0, junior females: 2, junior males: 4 [tennis only]). Importantly, given that rarely more than a total of 20 left-handed athletes were observed in annual rankings in squash (especially in senior females) and tennis (except for senior males), OR values need to be interpreted with caution in these sports in particular.

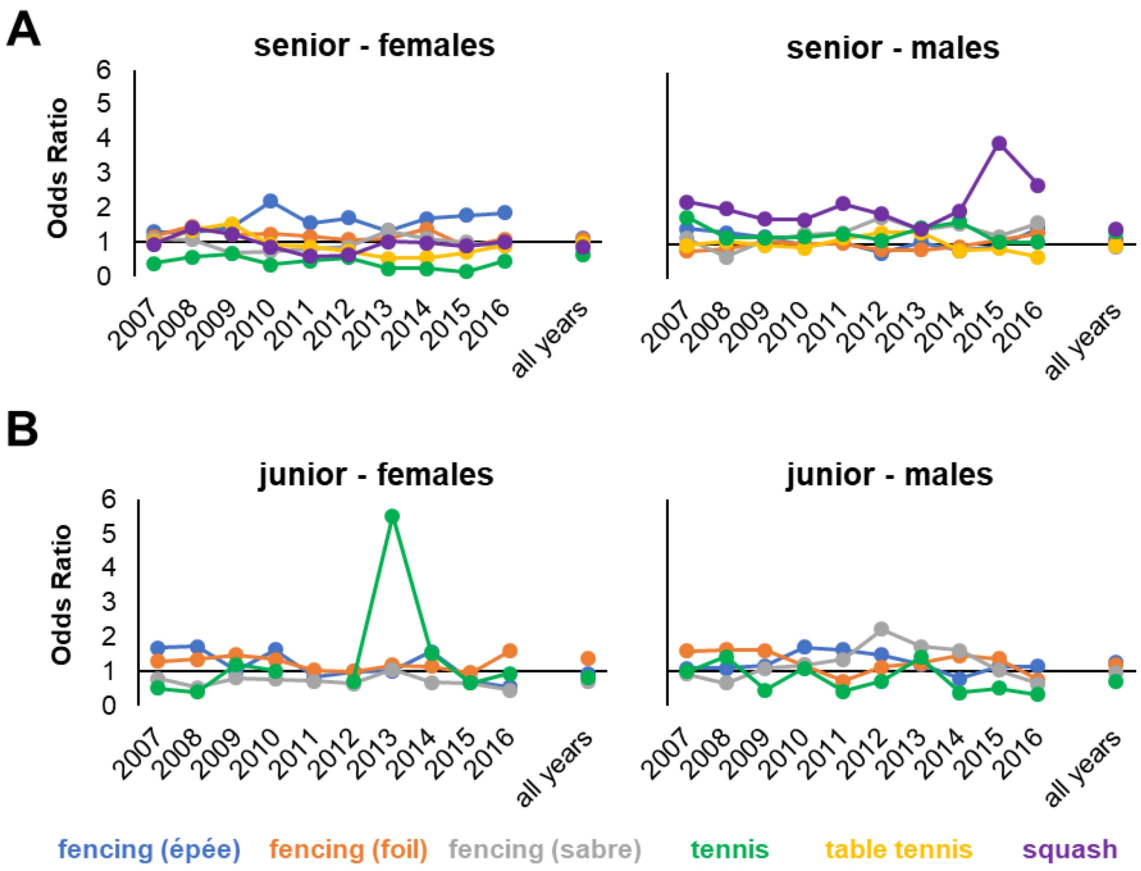

Supplementary Figure 2. Year-wise odds ratios (OR) representing the chances of right-handed athletes being born in the first vs. second half of a year compared to left-handed athletes being born in the first vs. second half of a year in a particular sport separately for (A) senior and (B) junior competition. OR values larger (smaller) than 1 indicate higher (lower) chances in right-handed as compared to left-handed athletes. If OR = 1, chances are equal in both groups. Note that OR is not shown for junior female tennis players in 2011 because none of the six left-handed players was born in the second half of the year.

SECTION B: MIXED CUT-OFF DATES (SEPTEMBER 1ST FOR UK ATHLETES; JANUARY 1ST FOR ATHLETES FROM OTHER NATIONS)

To account for the fact that, at the national level, UK athletes are grouped into cohorts based on September 1st as cut-off date and only at a later career stage, when progressing to the international competitive level, are grouped according to the internationally used cut-off date January 1st (e.g., Till et al., 2010; Kelly et al., 2020a; Kelly et al., 2020b), we re-run the analyses reported in the main text and in the supplementary Section A (see above) with UK athletes being classified into birth month and quartiles relative to September 1st (i.e. September = 1, October = 2 … August = 12; Quartile 1 = September-November … Quartile 4 = June-August). Birth months and quartiles of athletes from all other nations were kept classified according to January 1st (as in the main text) as we were not aware of any other nations employing cut-off dates different to that particular date. The results reported below follow the same structure as in the main text and the supplementary Section A. For details on data analyses please see the main text and Section A.

Birth Distribution

Senior Level. Across sports and athletes' sex, the 'classical' RAE distributional pattern of birth frequencies was not systematically skewed towards quartiles 1 and 2. As is illustrated in Supplementary Table 3, a RAE-like distribution of birth quartile frequencies was evident, to some extent, in female table tennis, sabre and épée as well as in male tennis. Similar to the results reported in the main text, effect size point estimates and 90% CI upper limits for these sports or disciplines were all below the conventional limit for a medium effect (Supplementary Table 3). Overall, even when considering mixed cut-off dates, there still is no systematic RAE present at the senior level.

Junior Level. Similar to the results reported in the main text, birth frequencies were skewed towards quartiles 1 and 2 in both female and male junior athletes (again except for female sabre; see Supplementary Table 3). The corresponding effect size point estimates, however, were located in the lower half of Cohen's (1988) conventional region of small effects (w = 0.1 to 0.3) and the upper limits of associated 90% CIs often did not exceed a value of w = 0.2. As for the results with a uniform cut-off date, the largest effect was found for male tennis (i.e., medium-sized effect associated with a gradual decrease of birth frequencies from Q1 to Q4).

Supplementary Table 3. Absolute frequencies and results from chi² goodness-of-fit tests (uniform distribution expected) related to relative age in senior and junior athletes.

			Relative Age							
**Age**	**Sex**	**Sport (Discipline)**	Q1	Q2	Q3	Q4	N/A	χ^2	*p*	*w* & 90% CI	*OR* & 95% CI
senior	female	fencing (épée)	165	173	165	126	0	8.55	.036	0.12 [0.02, 0.17]	1.31 [1.04, 1.65]
		fencing (foil)	177	164	146	141	0	5.26	.154	0.09 [0, 0.14]	1.26 [1.01, 1.57]
		fencing (sabre)	184	134	149	141	0	9.72	.021	0.13 [0.04, 0.18]	1.3 [1.05, 1.63]
		table tennis	160	114	131	95	7	18.26	< .001	0.19 [0.1, 0.26]	1.68 [1.31, 2.17]
		tennis	142	129	117	109	0	5.01	.171	0.1 [0, 0.16]	1.3 [1.02, 1.67]
		squash	142	126	137	127	14	1.37	.713	0.05 [0, 0.09]	1.12 [0.88, 1.42]
	male	fencing (épée)	180	155	163	153	1	2.78	.426	0.07 [0, 0.11]	1.18 [0.95, 1.46]
		fencing (foil)	158	177	147	155	0	3.04	.385	0.07 [0, 0.12]	1.02 [0.82, 1.27]
		fencing (sabre)	175	162	194	140	1	9.21	.027	0.12 [0.03, 0.17]	1.25 [1, 1.56]
		table tennis	119	102	113	98	3	2.61	.456	0.08 [0, 0.13]	1.21 [0.93, 1.59]
		tennis	126	132	115	85	0	11.43	.010	0.16 [0.06, 0.22]	1.48 [1.13, 1.95]
		squash	137	116	126	135	1	2.16	.541	0.06 [0, 0.11]	1.01 [0.8, 1.29]
junior	female	fencing (épée)	275	294	230	185	0	28.95	< .001	0.17 [0.11, 0.22]	1.49 [1.23, 1.79]
		fencing (foil)	270	254	225	186	0	17.46	.001	0.14 [0.07, 0.18]	1.45 [1.2, 1.75]
		fencing (sabre)	258	218	237	210	0	5.96	.114	0.08 [0, 0.12]	1.23 [1.02, 1.47]
		tennis	170	146	156	100	0	19.27	< .001	0.18 [0.1, 0.24]	1.7 [1.33, 2.18]
	male	fencing (épée)	322	294	279	236	0	13.68	.003	0.11 [0.05, 0.15]	1.36 [1.15, 1.61]
		fencing (foil)	275	301	236	212	0	18.45	< .001	0.13 [0.07, 0.18]	1.3 [1.08, 1.55]
		fencing (sabre)	285	269	263	218	0	9.56	.023	0.1 [0.03, 0.14]	1.31 [1.1, 1.56]
		tennis	215	184	141	110	0	39.61	< .001	0.25 [0.17, 0.31]	1.95 [1.55, 2.46]

Note: N/A = birth date not available, *w* = standardized Cohen's (1988) effect size and *OR* = odds ratio based on frequencies observed for Q1 and Q4 (see main text for details). df = 3 for all chi-square comparisons.

Birth Distribution and Handedness

When applied to the mixed cut-off date classifications, chi-square tests of independence between handedness and birth quartile reveal highly similar results (see Supplementary Table 4) compared to when the tests are applied to frequencies obtained from a uniform cut-off date (i.e. January 1st). Thus, the pattern of results reported in the main text does not change after the application of September 1st as cut-off date for UK athletes. Again, for both senior and junior athletes, analyses do not provide statistical support for the hypothesis that handedness and relative age are interrelated and they do not indicate that the RAE pattern in birth quartile distribution is more pronounced in right- than left-handers. As for the uniform cut-off date, the association found in female sabre fencers is in contrast to the latter prediction with birth distribution being more skewed towards Q1 in left-handed than right-handed fencers (Supplementary Figure 3A).

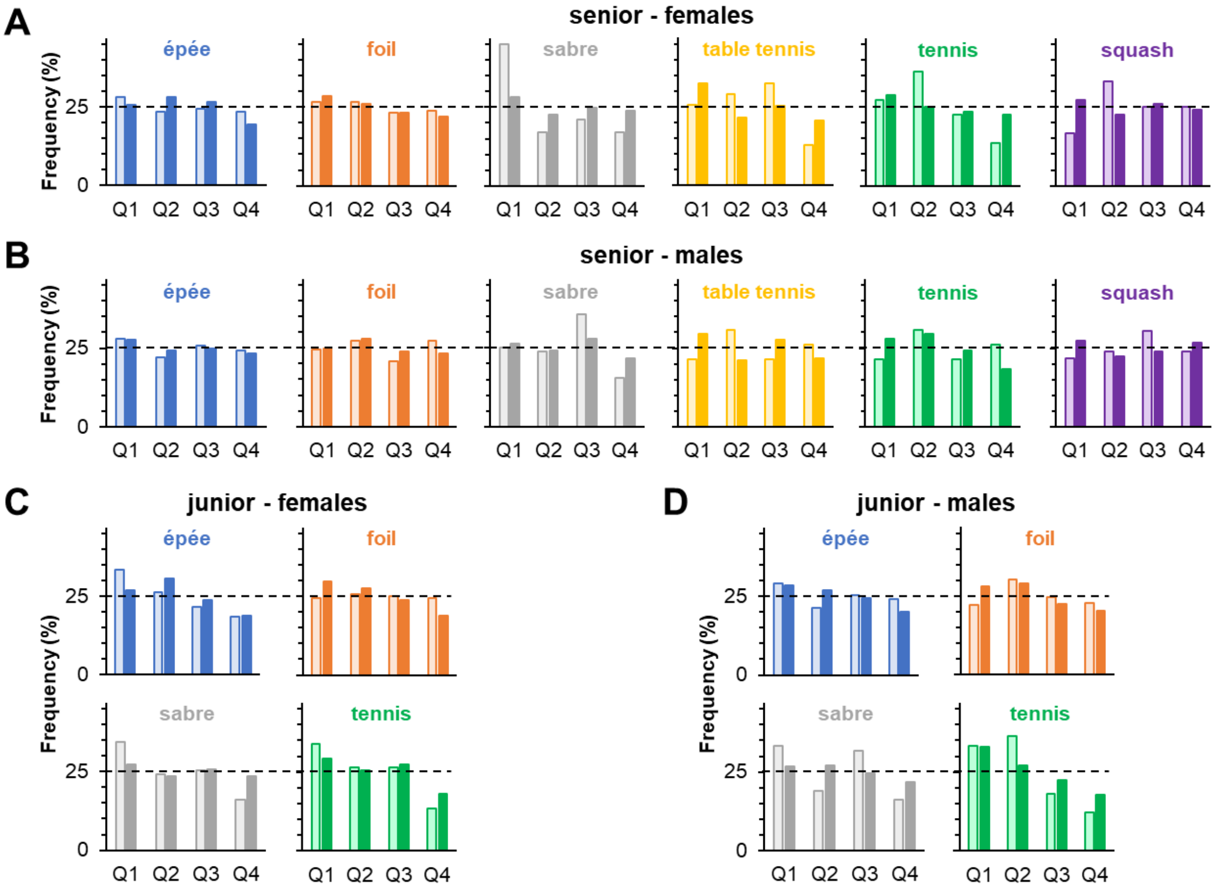

Supplementary Figure 3. Percentages of birth quartile (Q1-Q4) frequencies in left- (☐) and right-handers (■) separately for sport or discipline in (A) female and (B) male senior and in (C) female and (D) male junior athletes. Horizontal dashed lines indicate expected frequencies under the assumption of a uniform distribution.

Supplementary Table 4. Absolute frequencies and results from chi²-tests of independence between handedness and relative age in senior and junior athletes.

			**Left-Handed**				**Right-Handed**				**Chi²-test of independence**			
**Age**	**Sex**	**Sport (Discipline)**	Q1	Q2	Q3	Q4	Q1	Q2	Q3	Q4	χ^2	*p*	*w* & 90% CI	*OR* & 95% CI
senior	female	fencing (épée)	30	25	26	25	135	148	139	101	1.86	.601	0.05 [0, 0.1]	1.11 [0.62, 2.01]
		fencing (foil)	38	38	33	34	139	126	113	107	0.33	.954	0.02 [0, 0]	1.16 [0.69, 1.97]
		fencing (sabre)	32	12	15	12	152	122	134	129	8.53	.036	0.12 [0.02, 0.17]	0.44 [0.22, 0.89]
		table tennis	22	25	28	11	123	82	96	79	6.48	.091	0.12 [0, 0.18]	0.78 [0.36, 1.69]
		tennis	12	16	10	6	130	113	107	103	3.57	.312	0.08 [0, 0.14]	0.63 [0.23, 1.74]
		squash	6	12	9	9	133	110	128	118	3.13	.372	0.08 [0, 0.13]	1.69 [0.58, 4.89]
	male	fencing (épée)	38	30	35	33	142	125	128	120	0.30	.960	0.02 [0, 0]	1.03 [0.61, 1.74]
		fencing (foil)	40	45	34	45	118	132	113	110	1.43	.699	0.05 [0, 0.09]	1.21 [0.73, 1.99]
		fencing (sabre)	21	20	30	13	154	142	164	127	2.92	.404	0.07 [0, 0.11]	0.75 [0.36, 1.56]
		table tennis	23	33	23	28	94	67	88	69	7.01	.072	0.13 [0, 0.19]	1.66 [0.88, 3.12]
		tennis	14	14	18	12	112	118	97	73	1.87	.600	0.06 [0, 0.11]	1.32 [0.58, 3]
		squash	10	11	14	11	127	104	111	124	1.39	.708	0.05 [0, 0.1]	1.13 [0.46, 2.75]
junior	female	fencing (épée)	51	40	33	28	224	254	197	157	3.01	.390	0.06 [0, 0.09]	0.78 [0.47, 1.3]
		fencing (foil)	40	42	41	40	230	212	184	146	3.67	.300	0.06 [0, 0.1]	1.58 [0.97, 2.56]
		fencing (sabre)	30	21	22	14	228	197	215	196	3.35	.340	0.06 [0, 0.1]	0.54 [0.28, 1.05]
		tennis	18	14	14	7	152	132	142	93	1.00	.802	0.04 [0, 0.08]	0.64 [0.26, 1.58]
	male	fencing (épée)	61	45	53	51	261	249	226	185	3.58	.311	0.06 [0, 0.09]	1.18 [0.78, 1.79]
		fencing (foil)	47	64	52	48	227	236	183	164	2.95	.399	0.05 [0, 0.09]	1.41 [0.9, 2.22]
		fencing (sabre)	39	22	37	19	246	247	226	199	7.69	.053	0.09 [0, 0.13]	0.6 [0.34, 1.07]
		tennis	33	36	18	12	180	148	123	97	4.84	.184	0.09 [0, 0.14]	0.67 [0.33, 1.37]

Note: w = standardized Cohen's (1988) effect size and *OR* = odds ratio based on frequencies observed for right-handers in Q1 vs. Q4 relative to frequencies observed in left-handers in Q1 vs. Q4 (see main text for details). df = 3 for all chi-square comparisons.

Temporal stability of the association between birth month and birth frequency

As in Section A above, there is considerable variation in the relationship between birth month (relative to cut-off date of January 1st or September 1st) and birth frequency within and between sports/disciplines in both males and females age groups (Supplementary Figure 4). Overall, again the linear relationships appear more stable and consistent with RAE in junior (Supplementary Figure 4B) than senior competition (Supplementary Figure 4A).

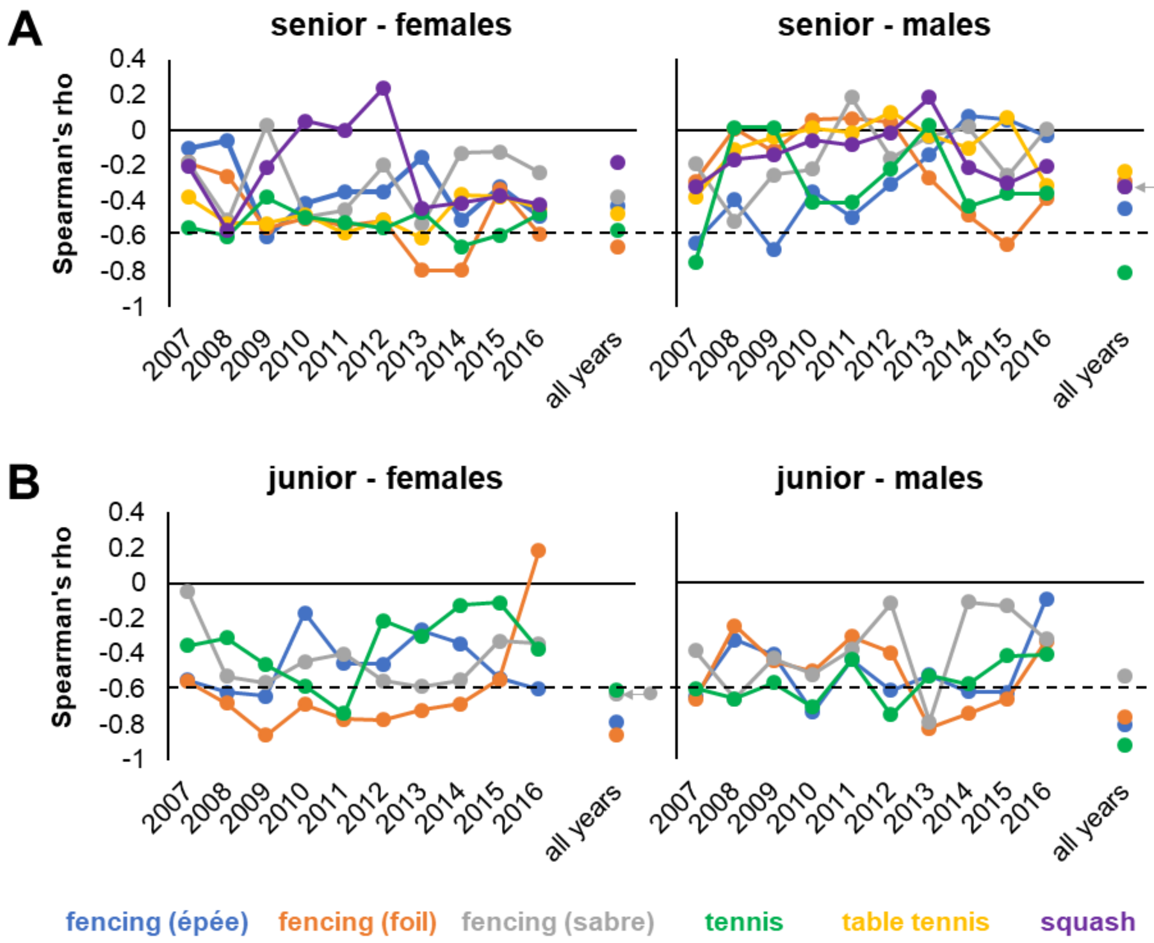

Supplementary Figure 4. Year-wise association between birth month relative to cut-off date and frequency of birth (Spearman's rho) for a particular sport separately for (A) senior and (B) junior competition when using mixed cut-off dates. Horizontal dashed lines indicate the critical value for Spearman's rho (±0.587) for $N = 12$, $\alpha = .05$ (two-tailed).

Temporal stability of the association between handedness and time of birth

Year-wise odds ratios are shown in Supplementary Figure 5 (for methodological details please see the corresponding part in Section A above). Overall, except for tennis in junior females 2013 (OR = 5.82;

Supplementary Figure 5B) odds ratios were relatively stable within sports across years. Note that the higher OR values in senior male squash reported in Section A for 2015 and 2016 are no longer evident (Supplementary Figure A). On rare occasions, OR values exceeded the value of 2 (senior females: 1, senior males: 3, junior females: 1, junior males: 1) or fell below the value of 0.5 (senior females: 6 [tennis only], senior males: 0, junior females: 3, junior males: 5 [tennis only]). As in Section A, it is important to keep in mind that, given that rarely more than a total of 20 left-handed athletes were observed in annual rankings in squash (especially in senior females) and tennis (except for senior males), OR values need to be interpreted with caution in these sports in particular.

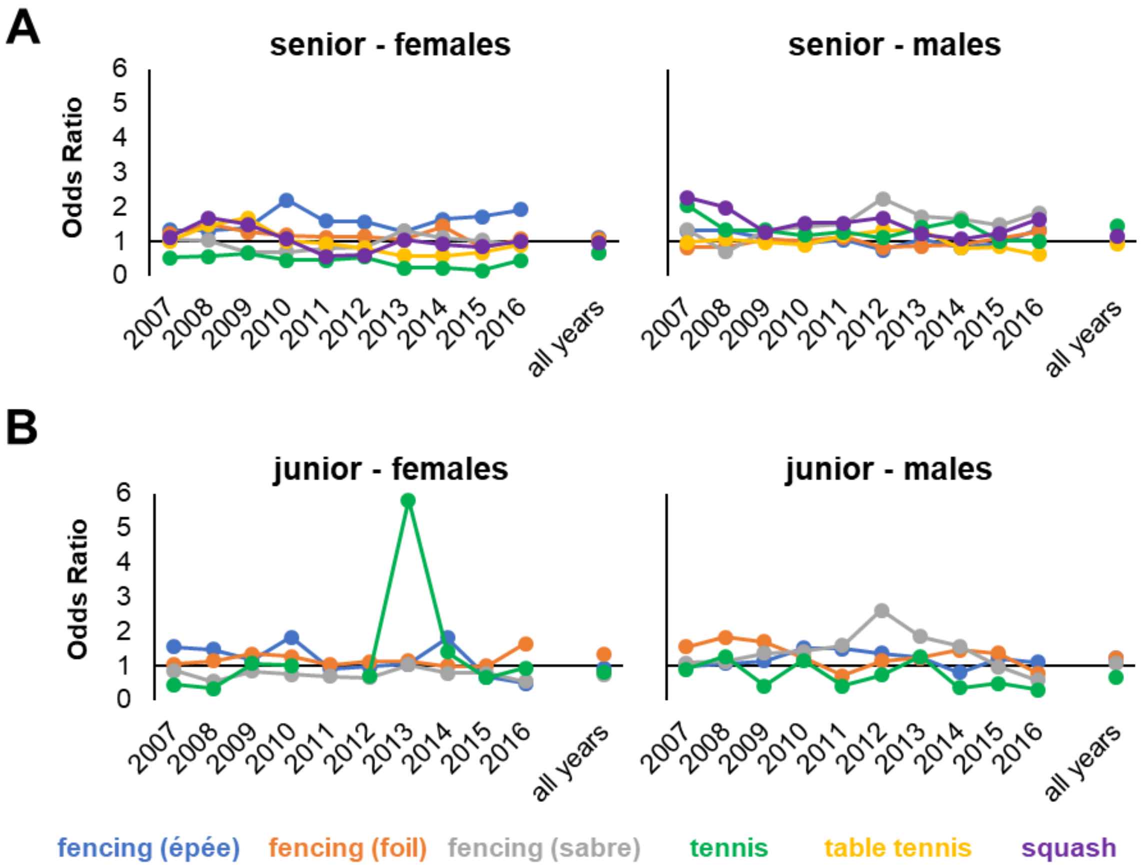

Supplementary Figure 5. Year-wise odds ratios (OR) representing the chances of right-handed athletes being born in the first vs. second half of a year compared to left-handed athletes being born in the first vs. second half of a year in a particular sport separately for (A) senior and (B) junior competition after application of mixed cut-off dates. OR values larger (smaller) than 1 indicate higher (lower) chances in right-handed as compared to left-handed athletes. If OR = 1, chances are equal in both groups. Note that OR is not shown for junior female tennis players in 2011 because none of the six left-handed players was born in the second half of the year.

References

Cohen, J. (1988). *Statistical power analysis for the behavioral sciences.* Hillsdale, NJ: Erlbaum.

Delorme, N., and Champely, S. (2015). Relative Age Effect and chi-squared statistics. *International Review for the Sociology of Sport* 50(6)**,** 740-746. doi: 10.1177/1012690213493104.

Kelly, A.L., Jackson, D.T., Taylor, J.J., Jeffreys, M.A., and Turnnidge, J. (2020a). “Birthday-banding” as a strategy to moderate the relative age effect: A case study into the England squash talent pathway. *Frontiers in Sports and Active Living* 2(145). doi: 10.3389/fspor.2020.573890.

Kelly, A.L., Wilson, M.R., Gough, L.A., Knapman, H., Morgan, P., Cole, M., et al. (2020b). A longitudinal investigation into the relative age effect in an English professional football club: exploring the ‘underdog hypothesis’. *Science and Medicine in Football* 4(2)**,** 111-118. doi: 10.1080/24733938.2019.1694169.

Loffing, F. (2016). Commentary: “How much is that player in the window? The one with the early birthday?” Relative age influences the value of the best soccer players, but not the best businesspeople. *Frontiers in Psychology* 7. doi: 10.3389/fpsyg.2016.00620.

Schorer, J., Roden, I., Büsch, D., and Faber, I. (2020). "Relative age effects are developmental! The necessity of looking at more than one time point," in *Relative age effects in sport: International perspectives,* eds. J.C. Dixon, S. Horton, L. Chittle & J. Baker. (New York: Routledge), 33-45.

Till, K., Cobley, S., Wattie, N., O'Hara, J., Cooke, C., and Chapman, C. (2010). The prevalence, influential factors and mechanisms of relative age effects in UK Rugby League. *Scandinavian Journal of Medicine & Science in Sports* 20(2)**,** 320-329. doi: 10.1111/j.1600-0838.2009.00884.x.
